# A Deep Learning-Based Method for Non-Destructive Estimation of Carbonate Carbon Storage in Biogenic Shells on Marine Engineering Materials

**DOI:** 10.3390/ma19040691

**Published:** 2026-02-11

**Authors:** Haonan Huang, Mengting Jia, Qiang Xu, Zhiqiang Cui, Junyu He

**Affiliations:** 1 Ocean College, Zhejiang University, Zhoushan 316021, China; 22534112@zju.edu.cn (H.H.); 19553716064@163.com (M.J.); 22334109@zju.edu.cn (Z.C.); jyhe@zju.edu.cn (J.H.); 2 Hainan Institute of Zhejiang University, Sanya 572025, China; 3 Ocean Academy, Zhejiang University, Zhoushan 316021, China

**Keywords:** marine engineering material surfaces, image-based quantitative assessment, hard-shelled organisms, carbonate carbon storage, non-destructive monitoring

## Abstract

**Highlights:**

**What are the main findings?**

- A non-destructive framework is developed to quantify shell carbonate carbon storage on marine engineering surfaces.
- An improved Mask Region-based Convolutional Neural Network (Mask R-CNN) enables automated identification and shell dimension extraction of barnacles and bivalves from in situ images.
- Image-derived shell dimensions show strong agreement with manual measurements (R^2^ = 0.95).

**What are the implications of the main findings?**

- Panel-scale carbonate carbon storage is estimated with errors below 15% under complex nearshore conditions.
- The proposed framework provides a non-destructive approach for comparative analysis and long-term monitoring of biofouling on different surface materials.
- Enables comparative quantification of biofouling and carbon storage across different materials.

**Abstract:**

Hard-shelled organisms colonizing marine engineering surfaces accumulate carbonate inorganic carbon in their shells, yet quantification typically relies on destructive sampling, hindering long-term monitoring. This study develops a deep learning-based, non-destructive framework to estimate shell carbonate carbon storage from in situ images. Panels of different surface materials were deployed in the nearshore waters of Liuheng Island (Zhoushan) and monitored for five months, yielding 90 panel images from June to October. An improved Mask R-CNN identified barnacles and bivalves and extracted shell dimensions, which were combined with allometric relationships and measured shell carbonate carbon fractions (12.07% for barnacles; 12.14% for bivalves) to estimate carbon storage. Peak colonization occurred on uncoated polyvinyl chloride (PVC) panels in September (~110 individuals per panel), corresponding to 1.061 g carbonate carbon per panel. The model achieved recall/precision of 0.86/0.89 under complex nearshore conditions; image-derived dimensions agreed with manual measurements (R^2^ = 0.95). Allometric models showed R^2^ of 0.82 (barnacles) and 0.90 (bivalves), and panel-scale estimation errors were <15%. The method enables non-destructive quantitative characterization and comparison of shell carbonate carbon storage across materials and exposure conditions for long-term monitoring.

## 1. Introduction

As one of the largest carbon sinks on Earth [1,2,3], the ocean plays a crucial role in regulating atmospheric CO_2_ concentrations, maintaining the stability of the climate system, and mitigating global warming [4,5]. Extensive observational and modeling studies have demonstrated that the ocean absorbs and stores a substantial proportion of anthropogenic carbon emissions through processes such as the transport of dissolved inorganic carbon, deep-ocean storage, and sedimentation [6]. Meanwhile, with the continuous intensification of coastal development, the spatial extent of marine engineering facilities—including ports, wharves, piles, and trestles—has expanded markedly in coastal zones [7], and their impacts on nearshore ecological processes have received increasing attention [8,9]. These marine structures provide stable substrates for benthic organisms, such as barnacles, mussels, and corals [10], promoting the formation of high-density biofouling communities and generating measurable amounts of shell-derived inorganic carbon at the scale of engineering surfaces [11]. However, the magnitude, spatial distribution, and quantitative assessment methods of this carbon storage remain insufficiently studied [12].

Within biofouling communities on marine engineering structures, hard-shelled organisms such as mussels and corals are often regarded as important contributors to inorganic carbon storage on artificial surfaces due to their strong shell mineralization capacity [13,14,15,16,17]. After death or detachment, their calcium carbonate shells can persist on engineering surfaces or in adjacent sediments over long timescales, forming relatively stable inorganic carbon storage pools [18,19]. In many nearshore marine engineering structures, however, sessile crustaceans such as barnacles often dominate in abundance [12]. Barnacles likewise produce calcium carbonate shells through biomineralization, and under high-density attachment conditions, their cumulative shell carbonate storage may be comparable to or even exceed that of bivalves. Although uncertainties remain regarding CO_2_ fluxes associated with barnacle calcification [20], and their long-term net carbon effects are still debated [21,22], the physical stability and persistence of barnacle shells are comparable to those of bivalves. Consequently, barnacles should not be overlooked in assessments of inorganic carbon storage at the scale of marine engineering surfaces. At present, studies on barnacle shell carbon content, volume-to-mass conversion, and long-term stability are very limited [23], which constrains the quantitative evaluation of carbonate carbon storage in hard-shelled biofouling communities.

Existing quantitative studies on biofouling on marine engineering facilities primarily rely on manual sampling or conventional image processing methods [24]. However, in nearshore environments characterized by high species diversity, pronounced size variability, and complex backgrounds, these approaches often fail to accurately identify attached organisms or estimate shell volumes, thereby limiting the precision of carbon storage assessments [25]. Advances in deep learning offer an effective pathway to overcome these limitations [26,27,28]. Rashid et al. [29] applied transfer learning to achieve automated detection of biofouling on tidal energy devices; Cui et al. [30] developed a multi-task deep learning framework capable of simultaneous classification, detection, and segmentation of marine organisms; and Sonmez et al. [31] achieved high-accuracy microalgae recognition by combining convolutional neural networks with traditional classifiers. These studies demonstrate the potential of deep learning for marine organism recognition. Nevertheless, high-precision segmentation and quantitative analysis of hard-shelled fouling organisms such as barnacles and bivalves remain challenging, particularly in harbor environments with multi-species overlap, variable illumination, and strong sediment interference [32]. Therefore, it is necessary to establish dedicated image datasets, enhance model robustness, and develop quantitative conversion frameworks linking image-derived features to shell carbonate carbon storage to support systematic assessments of artificial blue carbon.

Overall, existing research lacks an integrated methodological framework specifically designed for engineering-scale assessment of carbonate carbon storage associated with biofouling on marine engineering facilities, particularly under conditions involving complex species assemblages and shell carbonate carbon that is difficult to quantify using conventional approaches. To address these challenges, this study proposes a deep learning-based model for estimating carbonate carbon storage in the shells of hard-shelled organisms using periodically acquired in situ images. An instance segmentation model Mask Region-based Convolutional Neural Network (Mask R-CNN) [33] is employed to automatically identify barnacles and bivalves, and carbonate carbon storage is quantitatively estimated by integrating image-derived geometric features with allometric growth models. The resulting metric is intended to characterize carbon storage at the engineering surface scale and does not directly represent net atmospheric CO_2_ sequestration. This approach provides an efficient and non-destructive technique to support long-term carbon storage monitoring and enables automated evaluation of biofouling under different experimental conditions.

## 2. Marine Biofouling Image Dataset and Methods

### 2.1. Construction of the Image Recognition Dataset

#### 2.1.1. Acquisition of Raw Marine Biofouling Images

**Figure 1:**
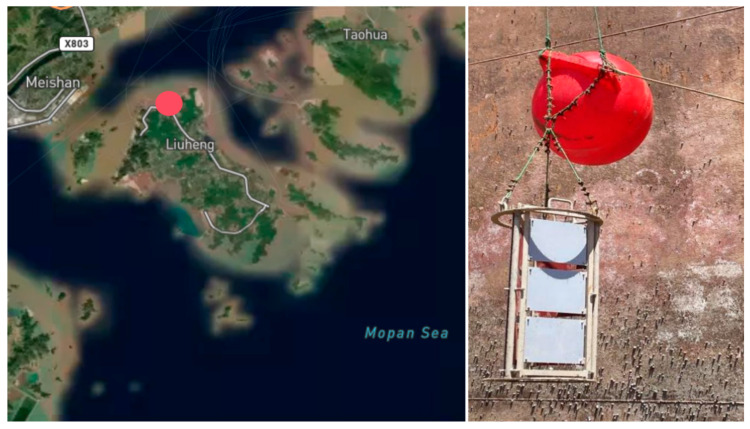
Location of the biofouling monitoring site and the experimental setup.

A shallow-sea in situ immersion experiment was conducted at a wharf berth located on the northern coast of Liuheng Island, Zhoushan, China (29°53′ N, 122°9′ E). As shown in Figure 1, the red marker denotes the geographic coordinate of the pier used for the in situ marine exposure experiments in this study. In accordance with the GB/T 5370–2007 [34] (Chinese national standard) standard and considering site-specific conditions, a multi-directional marine biofouling monitoring device was independently designed. The immersion depth of the panels was controlled at 1 m below the water surface.

Three groups of test panels, denoted as groups A, B, and K, were deployed, with three panels installed in each group. Detailed information on the panel configurations is provided in Table 1. Two experimental racks were installed simultaneously: one rack was replaced every month, while the other was subjected to continuous long-term immersion. Visual observations and image acquisition were performed monthly for both racks.

The immersion experiment was conducted from June to November 2025, during which the average seawater temperature was 25.72 °C, corresponding to the peak biological growth period in the East China Sea.

During image acquisition, the panels were rinsed with seawater before imaging and photographed vertically under natural lighting conditions. The entire imaging process was completed within 15 min to minimize the potential influence of panel exposure on the condition of the attached organisms.

#### 2.1.2. Manual Annotation and Data Augmentation

**Figure 2:**
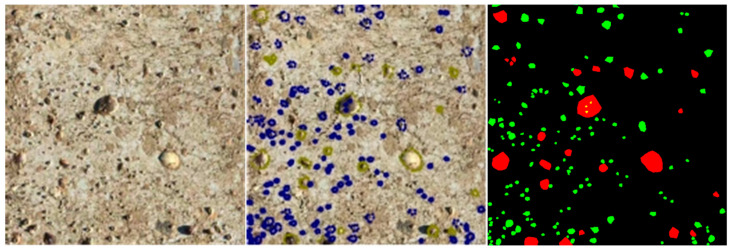
Pixel-level annotations and corresponding masks were generated using LabelMe.

Pixel-level instance segmentation annotations were performed for major hard-shelled fouling organisms, including barnacles and bivalves, using the LabelMe tool [35]. Based on these annotations, a benchmark dataset comprising 90 high-quality labeled images was constructed, as illustrated in Figure 2.

To enhance the model’s ability to recognize small-scale fouling organisms under complex nearshore conditions, multiple data augmentation strategies were applied [36], as summarized in Table 2. The original images were partitioned using a fixed sliding-window cropping strategy with a size of 512 × 512 pixels to match the unified input resolution of the model while avoiding the loss of small-scale biological features caused by global image resizing. After cropping, the training samples were randomly subjected to geometric transformations, illumination and color perturbations, and small-object enhancement operations, while maintaining spatial consistency between the images and their corresponding instance masks. After data augmentation, the number of training images was expanded from the original 90 images to approximately 8000, thereby improving model stability and generalization performance across multiple scales and background conditions.

### 2.2. Deep Learning-Based Marine Organism Recognition Method

To achieve non-destructive quantitative identification and measurement of biogenic shells on the surfaces of marine engineering materials, this study adopts an existing open-source Mask R-CNN-based instance segmentation framework to automatically detect and segment attached organisms. This method outputs instance-level segmentation masks on the basis of object detection, providing the foundation for subsequent extraction of shell geometric parameters and the estimation of carbonate carbon storage.

#### 2.2.1. Model Architecture

Mask R-CNN introduces an instance-level mask prediction branch on the basis of Faster R-CNN, enabling object detection, classification, and pixel-level segmentation to be performed within a unified framework. This architecture is well suited for applications that require accurate delineation of individual biological instances and extraction of their geometric information.

The overall model architecture consists of three main components: a feature extraction backbone network, a Region Proposal Network (RPN), and a mask prediction branch. In this study, the backbone network adopts a ResNet-50 [37,38] architecture combined with a Feature Pyramid Network (FPN). By integrating multi-scale feature representations, the FPN enhances the model’s ability to represent small-scale targets while maintaining computational efficiency.

**Figure 3:**
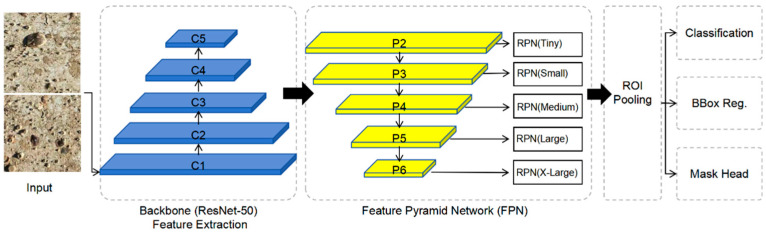
Architecture of the instance segmentation model based on Mask R-CNN.

Figure 3 presents a schematic diagram of the Mask R-CNN-based instance segmentation framework used in this study, illustrating the overall workflow and relationships among key stages, including feature extraction, candidate region generation, and instance mask prediction.

#### 2.2.2. Model Adaptation Strategy

Hard-shelled fouling organisms in nearshore marine environments are typically characterized by small individual sizes, dense spatial distribution, and local occlusion. In response to these scene characteristics, this study focuses on stable detection of small-scale targets and complete representation of instance contours through appropriate model configuration and usage strategies.

Within the RPN, the scales and densities of anchor boxes are carefully configured to increase the probability that true targets are effectively covered under high-density fouling conditions, thereby reducing missed detections of small-scale biological instances. These settings are primarily intended to meet the requirements of engineering applications for instance completeness, rather than to pursue extreme optimization of detection accuracy.

During instance segmentation training, a hybrid loss function combining Cross-Entropy Loss and Dice Loss is adopted to balance training stability and segmentation quality for irregular shell contours. The cross-entropy loss constrains the convergence of the overall pixel-wise classification process, while the Dice loss mitigates class imbalance between foreground and background regions, thereby improving segmentation completeness in areas with blurred boundaries or local occlusion. This type of hybrid loss function has been widely applied in instance segmentation and medical image segmentation tasks [39], and its effectiveness has been demonstrated in numerous studies.

During both model training and inference, an input size of 512 × 512 pixels is consistently used. For high-resolution original panel images, a sliding-window strategy with the same window size is applied for patch-wise inference, and the prediction results are subsequently merged in the original image coordinate system. This strategy avoids information loss caused by global image resizing and enhances the reliability of small-scale fouling organism recognition under complex background conditions. The relevant model parameters are summarized in Table 3.

#### 2.2.3. Model Evaluation Methods

To quantitatively evaluate the recognition and segmentation performance of the instance segmentation model on biofouling images, this study employs commonly used evaluation metrics, including mean average precision (mAP), intersection over union (IoU), and the Dice coefficient, with comparisons conducted against manually annotated results. The model achieves stable segmentation accuracy for both barnacles and bivalves, enabling the segmentation outputs to serve as a reliable basis for subsequent extraction of biological geometric features and estimation of carbonate carbon storage.

Intersection over Union (IoU) is used to measure the degree of overlap between the predicted mask and the manually annotated ground-truth mask, and is defined as follows:

(1) $$IoU=\frac{TP}{TP+FP+FN}$$

where TP, FP, and FN denote the numbers of true positive, false positive, and false negative pixels, respectively.

The Dice coefficient is further used to evaluate the consistency of the segmentation results, and is defined as follows:

(2) $$Dice=\frac{2\times TP}{2\times TP+FP+FN}$$

The mean average precision (mAP) is defined as the arithmetic mean of the average precision (AP) values calculated for each target class. In this study, mAP is reported following the COCO evaluation protocol, where AP is averaged over multiple IoU thresholds ranging from 0.50 to 0.95 with a step size of 0.05. The mAP is defined as follows:

(3) $$mAP=\frac{1}{N}\sum_{i=1}^{N}AP_{i}$$

where N denotes the number of target categories included in the evaluation, and $AP_{i}$ denotes the average precision for category i, computed over different IoU thresholds.

### 2.3. Calculation of Shell Carbonate Carbon Storage Based on Recognition Results

Based on the recognition results and allometric growth theory [40], this study establishes a quantitative framework for calculating shell carbonate carbon storage in hard-shelled organisms (barnacles and bivalves).

The basic formula for estimating the shell carbonate carbon storage of an individual organism is given as follows:

(4) $$C_{\mathrm{shell}}=W_{\mathrm{shell}}\times C_{\mathrm{carbonate}}=C_{\mathrm{carbonate}}\times \alpha \times {\left(\frac{\pi }{4}\cdot L\cdot B\right)}^{\beta }$$

where $C_{\mathrm{shell}}$ denotes the carbonate carbon mass corresponding to an individual shell (unit: g), $W_{\mathrm{shell}}$ represents the dry weight of the shell (unit: g), and $C_{\mathrm{carbonate}}$ is the carbon content fraction. The carbon content fraction was determined by selecting more than ten dried shell samples, mixing them by species, and measuring the inorganic carbon content using an Isoprime 100 isotope ratio mass spectrometer. The final value was obtained as the average of three repeated measurements.

As image-based recognition can only provide top-view two-dimensional geometric features of individual organisms, the shell dry weight cannot be measured directly. Therefore, based on allometric growth theory, the shell dry weight is expressed as a function of the projected area of the individual, where $\frac{\pi }{4}\cdot L\cdot B$ approximately represents the equivalent projected area of the shell, and L and B denote the projected length and projected width of the shell (unit: cm), respectively. The parameters α and β are species-specific scaling coefficients and allometric exponents, respectively. These parameters were determined by fitting shell length, shell width, and shell dry weight data—measured using a vernier caliper and an electronic balance—via a nonlinear least-squares method.

## 3. Results and Analysis

### 3.1. In Situ Observation Results of Marine Biofouling

**Figure 4:**
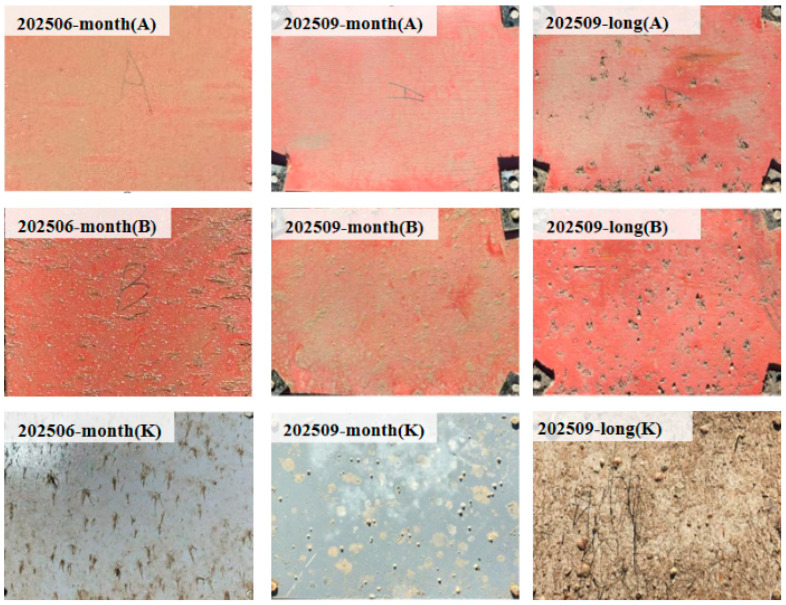
Photographs of the panels at the initial and final stages of the in situ immersion experiment. Long denotes long-term immersion, and month denotes monthly replacement.

As shown in Figure 4, only a small number of early-stage fouling organisms were observed on the panel surfaces during the initial phase of the in situ immersion experiment (June 2025). By the end of the observation period (September 2025), the panel surfaces had developed multi-species fouling communities composed of barnacles, bivalves, algae, and other organisms. These communities were characterized by high attachment density, heterogeneous spatial distribution, and pronounced variability in individual sizes. Under natural marine environmental conditions, fouling organisms often coexist with background biofilms, shadows, and illumination variations, resulting in complex object boundaries and increasing the difficulty of stable quantitative analysis using manual counting or conventional image processing methods.

Clear differences were observed between monthly replaced panels and long-term immersed panels in terms of fouling accumulation and retention characteristics, providing comparative samples for subsequent image-based quantitative analyses. The in situ seawater temperature was also recorded during each sampling event (as shown in Table 4), providing basic environmental information for subsequent analysis of biofouling dynamics.

### 3.2. Deep Learning-Based Recognition of Marine Hard-Shelled Organisms

#### 3.2.1. Model Performance and Recognition Results

The augmented dataset was split into training and testing sets at a ratio of 8:2. The split was performed based on the original images to avoid information leakage caused by cropped image patches appearing in different datasets. Under complex background conditions typical of nearshore waters in the East China Sea—characterized by high turbidity, dense biofouling, and a high proportion of small targets—the model demonstrated stable recognition performance. The evaluation results on the test set are summarized in Table 5.

**Figure 5:**
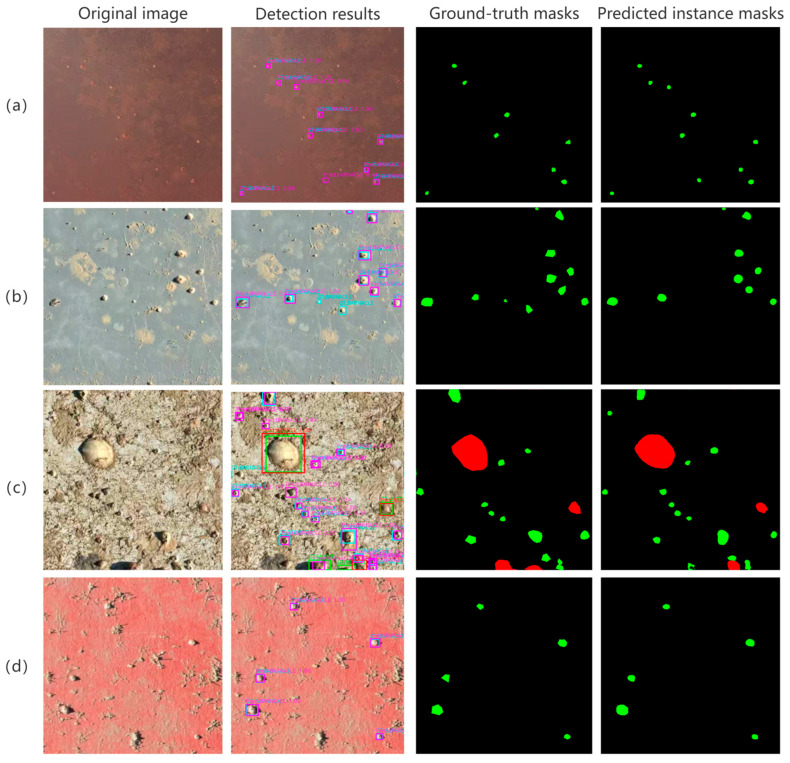
Representative instance segmentation results of barnacles and bivalves under different background conditions. Green and red regions indicate barnacles and bivalves, respectively; ground-truth and detected instances are shown by different colored bounding boxes. (**a**) PVC panel with non-uniform illumination; (**b**) PVC panel with a relatively clean background; (**c**) PVC panel with a complex background and dense attachment; (**d**) red-coated panel under normal illumination.

The model achieved scores exceeding 0.85 for both precision and small-object recall, indicating effective identification of most barnacle and bivalve targets in the images. Additionally, the IoU and Dice coefficients achieved 0.65 and 0.78, respectively, indicating a good pixel-level agreement between the segmentation results and manual annotations. In contrast, the mAP value was 0.54, reflecting a certain degree of misidentification in regions with complex backgrounds, dense attachment, and overlapping individuals. On panels with relatively clean backgrounds and more uniform organism distributions, the segmentation performance improved markedly. As shown in Figure 5d, the Dice coefficient increased to 0.91, with an mAP of 1.00. This indicates that the reduction in mAP is primarily attributable to recognition uncertainty in locally complex scenes, rather than to an overall deficiency in the model’s segmentation capability.

Figure 5 presents representative recognition results of the model under different background complexities and surface types. Overall, the model is capable of stably identifying hard-shelled organisms such as barnacles and bivalves under a wide range of complex imaging conditions. The segmented contours show good agreement with manual annotations, with only a limited number of instance merging and small-object missed detections occurring in regions with extremely dense attachment.

#### 3.2.2. Consistency Analysis Between Recognition Results and Manual Measurements

**Figure 6:**
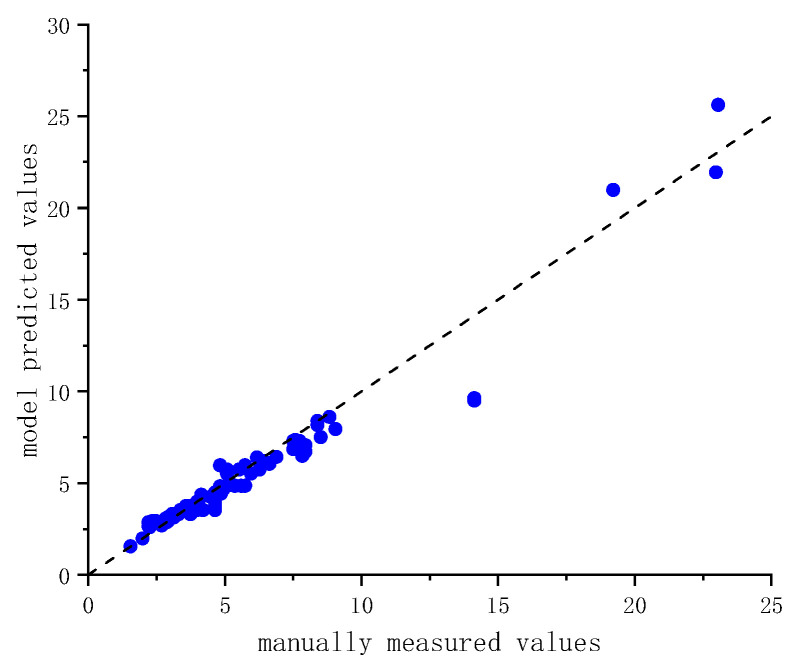
Comparison between image-derived and manually measured shell dimensions, including shell length (mm) and shell width (mm).

To evaluate the accuracy of the model in identifying the sizes of hard-shelled organisms, 83 individuals of barnacles and bivalves were randomly selected from the in situ immersed panels. Their shell length and shell width were measured manually and compared with the corresponding model-derived results, as shown in Figure 6.

The shell length and shell width obtained from model-based recognition show strong agreement with the corresponding manual measurements, with an overall correlation coefficient of R^2^ = 0.95, indicating that the model can stably extract geometric features with accuracy comparable to manual measurements. A small number of deviating data points are mainly observed in the larger size range. This can be attributed to the absolute amplification of boundary errors during the segmentation of large shells, as well as the effects of local occlusion or incomplete contours on geometric parameter extraction. In addition, the relatively low proportion of large-sized samples in the dataset increases the uncertainty in this range. Overall, the number of such deviating samples is limited, and the associated errors remain within an acceptable range. Considering that medium- to large-sized individuals contribute dominantly to total carbonate carbon storage, and that the allometric growth model is relatively insensitive to small-scale dimensional errors, the segmentation results are sufficient to meet the requirements for carbonate carbon quantification at the engineering scale.

### 3.3. Calculation of Shell Carbonate Carbon Storage

#### 3.3.1. Determination of Model Parameters for Shell Carbonate Carbon Storage

**Figure 7:**
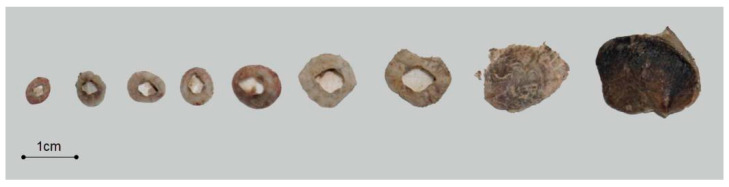
Representative samples of barnacles and bivalves.

To establish a shell carbonate carbon storage calculation model suitable for this study, different parameter determination strategies were adopted for barnacles and bivalves to account for differences in sample size and morphological characteristics between the two organism groups (Figure 7).

**Figure 8:**
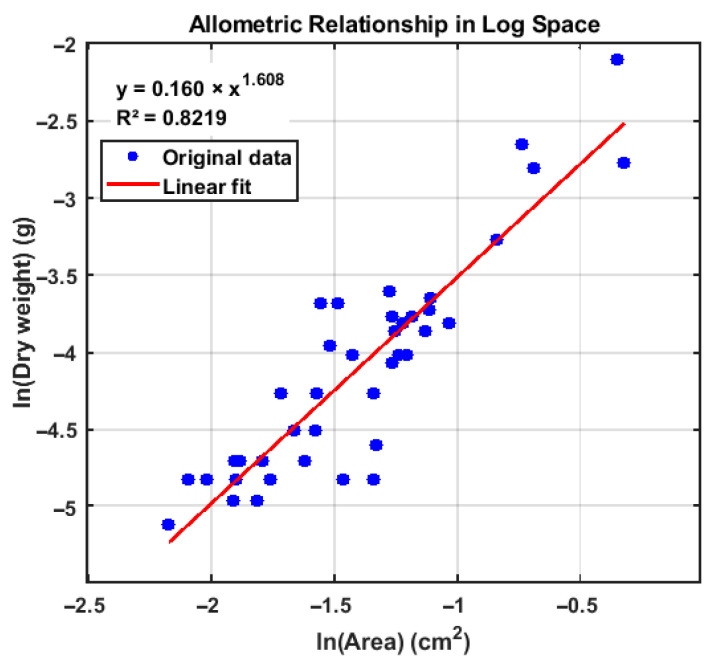
Fitting results between the attachment area and the shell dry weight.

For barnacles, the model parameters were obtained through fitting, yielding a coefficient of determination of R^2^ = 0.82 (Figure 8). The results indicate that the relationship between shell dimensions and dry weight can be well described by a power-law function, and that the current accuracy of two-dimensional size recognition is sufficient to meet the requirements for carbonate carbon storage estimation.

For bivalves, due to the limited number of available samples, allometric growth parameters of a closely related species with shell morphological characteristics similar to those of the bivalves observed on the panels were adopted from the literature and subsequently validated using the samples collected in this study. The validation results show that the transferred model achieved a coefficient of determination of R^2^ = 0.90 (Table 6), indicating that this parameter set is applicable at the scale of the present study and can be used for the estimation of shell carbonate carbon storage in bivalves.

#### 3.3.2. Calculation of Carbonate Carbon Storage Based on Recognition Results

The carbonate carbon mass fractions of barnacles and bivalves were determined to be 12.07% and 12.14%, respectively, using an Isorime100 isotope ratio mass spectrometer. This parameter is primarily used for the relative comparison of carbonate carbon storage among different material surfaces, and its uncertainty does not alter the observed trends in carbon storage variation among the different panels. By combining these values with individual dry weights estimated from the allometric growth models, a conversion from two-dimensional image-derived size parameters to three-dimensional shell dry weight and carbonate carbon storage can be achieved. This enables the establishment of a carbonate carbon storage calculation framework for hard-shelled organisms that is suitable for long-term, non-destructive monitoring.

Taking Figure 5c as an example, instance segmentation was first applied to the panel image to extract pixel-level geometric information. Based on the actual panel dimensions (25 cm × 35 cm), the projected length (L) and width (B) were scaled accordingly and substituted into the corresponding models to estimate shell dry weight and carbonate carbon storage, thereby obtaining the total carbonate carbon storage of hard-shelled organisms within the image.

Based on the calculation results presented in Table 7, the total carbonate carbon storage of the 21 hard-shelled organism individuals in Figure 5c was estimated to be 0.6854 g. Compared with the measured value of 0.6037 g, the relative error was less than 15%. This result indicates that, under the current levels of recognition accuracy and parameter reliability, image-based estimation of carbonate carbon storage can reasonably reflect the actual carbon storage at the panel scale. The proposed approach, therefore, provides a simple and efficient tool for material performance evaluation, refined carbonate carbon accounting, and the planning and deployment of marine engineering facilities.

### 3.4. Panel-Scale Shell Carbonate Carbon Storage Under In Situ Conditions

#### 3.4.1. Monthly Variations in Carbonate Carbon Storage

Based on the recognition results obtained from the monthly replaced panels, the abundance of hard-shelled organisms and their associated carbonate carbon storage on group K panels were analyzed for different months under the same exposure duration. As no hard-shelled organisms were detected on panels from groups A and B within a single month, these panels were excluded from the comparative analysis.

**Figure 9:**
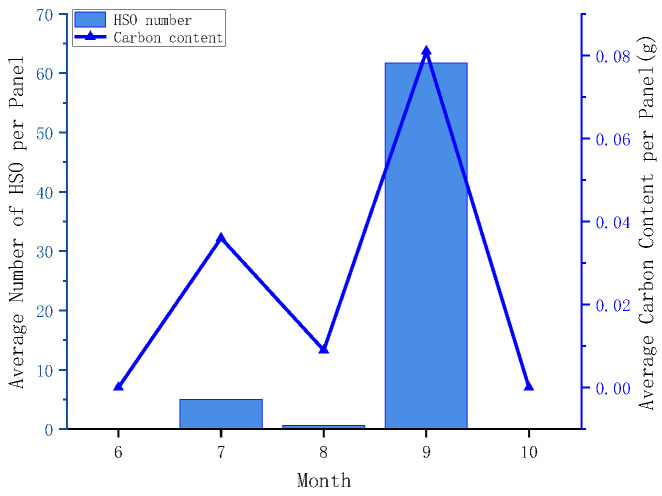
Monthly variations in the abundance of hard-shelled organisms (HSOs) and carbonate carbon storage on group K panels under monthly replacement conditions.

The results reveal pronounced monthly differences in both biofouling abundance and carbonate carbon storage (Figure 9). No hard-shelled organism attachment was observed in June or November, which can be attributed to relatively low seawater temperatures (<25.55 °C) [42,43]. Initial colonization occurred in July, when temperatures exceeded 25.55 °C, with an average carbonate carbon storage of approximately 0.036 g per panel. By September, when temperatures rose above 27.51 °C, the average carbonate carbon storage increased to 0.081 g per panel. In August, despite elevated seawater temperatures, a temporary decline in attachment intensity was observed. This reduction is likely related to typhoon-induced changes in water flow [44,45], indicating that hard-shelled organism attachment is influenced by coupled environmental factors rather than temperature alone.

Overall, the proposed non-destructive detection method is capable of distinguishing variations in colonization intensity and carbonate carbon storage on marine engineering surfaces at a monthly timescale, while remaining sensitive to short-term environmental fluctuations.

#### 3.4.2. Comparison of Carbonate Carbon Storage Among Different Surface Materials

**Figure 10:**
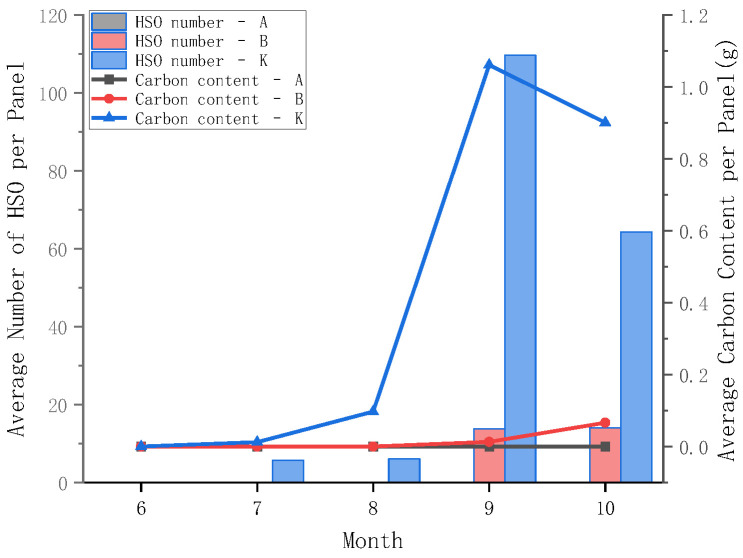
Variations in the abundance of hard-shelled organisms (HSOs) and carbonate carbon storage on panels with different surface materials under long-term immersion conditions.

Based on the recognition results obtained from the long-term immersion setup, the abundance of hard-shelled organisms and the average carbonate carbon storage per panel were compared among panels with three different surface materials (groups A, B, and K) under continuous exposure conditions. The results show that the differences among the three surface materials became progressively amplified over time (Figure 10). Group K exhibited the highest cumulative carbon storage capacity, with values markedly higher than those observed under monthly replacement conditions.

During July–August (25.55–27.22 °C), early-stage attachment occurred on group K panels, with average abundances of 5 and 6 individuals per panel and corresponding carbonate carbon storage values of 0.012 and 0.097 g per panel, respectively, without the monthly declines observed for replaced panels. By September, the abundance and carbonate carbon storage on group K panels increased sharply to 110 individuals per panel and 1.061 g per panel, respectively. In contrast, initial colonization on group B panels was first observed during this period (14 individuals per panel), with a much lower carbonate carbon storage of only 0.013 g per panel. After seawater temperature decreased in October, the abundance on group K panels declined to 64 individuals per panel, while carbonate carbon storage remained relatively high at 0.901 g per panel. This indicates that, under long-term immersion conditions, carbonate carbon storage is strongly influenced by cumulative effects and shell retention [46,47], and does not necessarily vary synchronously with the contemporaneous abundance observed on monthly replaced panels.

When antifouling coatings were present on the panel surfaces, attachment of hard-shelled organisms was effectively suppressed [48,49]. Both groups A and B exhibited substantially lower organism abundance and carbonate carbon storage than group K. Group B showed limited carbonate carbon contribution during the later stages of exposure, whereas group A maintained an approximately “zero-carbon-storage” state throughout the experiment. Under relatively low-flow conditions, panels coated with SeaQuantum Pro U exhibited higher hard-shelled organism abundance and carbonate carbon storage than those coated with SeaForce Active.

Based on the comprehensive analysis of marine biofouling images presented in this study, the proposed deep learning-based method for quantifying carbonate carbon storage is demonstrated to be fast, efficient, non-destructive, and convenient. The method is capable of capturing variations in colonization intensity across different temporal windows and distinguishing differences in carbonate carbon storage performance among surface materials under long-term service conditions. In the future, this approach can be extended to in situ, non-destructive, and continuous underwater monitoring, providing a quantitative and comparable technical tool for the design of artificial surfaces and materials with targeted carbon storage functions.

## 4. Conclusions

This study addressed the quantification of carbonate carbon storage associated with hard-shelled organisms on marine engineering test panels and developed and validated an integrated technical framework combining real-sea exposure experiments, image-based recognition, and carbonate carbon storage estimation, with validation conducted under controlled nearshore exposure conditions using periodically retrieved test panels. The main conclusions are summarized as follows:

(1) A five-month shallow-sea immersion experiment was conducted, yielding 90 biofouling images. Barnacle colonization began when seawater temperature exceeded 25.55 °C. In September, when the maximum temperature reached 27.77 °C, the abundance of hard-shelled organisms on group K panels peaked at 110 individuals per panel. Panels coated with antifouling materials (groups A and B) exhibited substantially lower organism abundance than the uncoated K panels.
(2) The Mask R-CNN-based instance segmentation model achieved stable recognition of hard-shelled organisms, including barnacles and bivalves, under complex nearshore conditions in the East China Sea characterized by high turbidity, dense attachment, and pronounced scale variability. On the test set, the model attained a recall of 0.86 and a precision of 0.89. The shell length and width extracted by the model showed strong agreement with manual measurements (R^2^ = 0.95), providing reliable geometric inputs for subsequent carbon storage calculations.
(3) By integrating image-derived geometric features with allometric growth relationships, a quantitative pathway was established and validated to estimate three-dimensional shell carbonate carbon storage of barnacles and bivalves from two-dimensional projection information. The fitted allometric models achieved coefficients of determination of 0.82 for barnacles and 0.90 for bivalves.
(4) Significant differences were observed among artificial surface materials in terms of hard-shelled organism attachment intensity and carbonate carbon storage capacity. Under long-term immersion conditions, uncoated PVC panels exhibited markedly higher attachment density and carbonate carbon storage per panel than antifouling-coated panels, with peak abundances of 110 and 14 individuals per panel, respectively. Under relatively static conditions, SeaQuantum Pro U-coated panels exhibited higher hard-shelled organism abundance and carbonate carbon storage than SeaForce Active-coated panels.

## Figures and Tables

**Table 1:** Sample information.

Panel Group	Substrate Material	Coating Type
Group K test panels	polyvinyl chloride (PVC)	None
Group A test panels	Steel	JOTUN Seaforce Active (140 µm, Jotun A/S, Norway)
Group B test panels	Steel	JOTUN SeaQuantum Pro U (240 µm, Jotun A/S, Norway)

**Table 2:** Data augmentation strategies and their primary purposes.

Augmentation Category	Specific Operations	Primary Purpose	Target Objects
Geometric transformations	Random flipping, 90° rotation, random scaling	Enhance model robustness to variations in viewing angles and object scales.	All samples
Illumination and color augmentation	Brightness/contrast perturbation, HSV jittering, CLAHE	Simulate natural lighting variations and enhance contrast between organisms and background	All samples
Image cropping	512 × 512 px sliding-window cropping	Preserve original pixel information, increase the relative scale of small targets, and reduce background interference.	Fouling regions in all samples
Small-object enhancement	Gaussian noise, blurring, copy–paste of small targets	Increase the diversity of small-object samples and improve recall	Barnacles and bivalves

**Table 3:** Key parameter settings for Mask R-CNN training and inference.

Parameter	Value/Setting	Description
Model	Mask R-CNN	Based on a mature deep learning framework
Backbone network	ResNet50–FPN	Default architecture of the framework, not modified
Input image size	512 × 512	Input after image cropping
Training epochs	300	Early stopping enabled with a Monitored on validation loss with a patience of 30 epochs
Optimizer	AdamW	—
Learning rate	2 × 10^−4^ (mask branch)/ 1 × 10^−4^ (others)	Branch-specific learning rates
Weight decay	1 × 10^−4^	AdamW parameter
Learning rate scheduling	CosineAnnealingLR + ReduceLROnPlateau	Combined scheduling strategy
Loss function	Hybrid loss	Cross-entropy + Dice
Class weights	[1.0, 3.0, 2.0]	background/mussel/barnacle
Data augmentation	Geometric + appearance + copy–paste	Randomly applied

**Table 4:** Seawater Temperature.

**Date**	4 June	1 July	1 August	2 September	9 October	6 November
**Temperature (°C)**	24.5	25.55	27.22	27.51	27.77	21.81

**Table 5:** Performance metrics of the model.

mAP	IoU	Dice	Small-Object Recall	Precision
0.54	0.65	0.78	0.86	0.89

**Table 6:** Allometric growth parameters used for carbonate carbon storage estimation.

Organism Group	α	β	R^2^	Parameter Source
Barnacles	0.1597331	1.60845	0.82	This study
Bivalves	0.0622163	2.54634	0.90	[41]

**Table 7:** Calculated shell carbonate carbon storage for organisms in Figure 5c.

No.	Species	L (mm)	B (mm)	Attachment Area (cm^2^)	Shell Dry Weight (g)	Carbonate Carbon Storage (g)
1	Bivalves	25.62	21.94	4.4147	5.4586	0.6627
2	Barnacle	6.49	5.52	0.2814	0.0208	0.0025
3	Barnacle	4.86	4.03	0.1538	0.0079	0.0009
4	Barnacle	4.48	3.98	0.1400	0.0068	0.0008
5	Barnacle	3.98	3.58	0.1119	0.0047	0.0006
6	Bivalves	7.51	6.72	0.3964	0.0118	0.0014
7	Barnacle	4.86	4.25	0.1622	0.0086	0.0010
8	Barnacle	3.75	3.36	0.0990	0.0039	0.0005
9	Barnacle	2.87	2.91	0.0656	0.0020	0.0002
10	Barnacle	3.09	2.91	0.0706	0.0022	0.0003
11	Barnacle	9.63	7.07	0.5347	0.0584	0.0070
12	Barnacle	5.74	4.03	0.1817	0.0103	0.0012
13	Bivalves	7.95	6.05	0.3778	0.0104	0.0013
14	Barnacle	3.58	3.09	0.0869	0.0031	0.0004
15	Barnacle	3.53	2.69	0.0746	0.0025	0.0003
16	Barnacle	4.86	4.03	0.1538	0.0079	0.0009
17	Barnacle	3.75	3.13	0.0922	0.0035	0.0004
18	Barnacle	6.49	3.31	0.1687	0.0091	0.0011
19	Barnacle	4.64	4.48	0.1633	0.0087	0.0010
20	Barnacle	3.09	2.69	0.0653	0.0020	0.0002
21	Barnacle	3.75	3.13	0.0922	0.0035	0.0004

## Data Availability

The original contributions presented in this study are included in the article. Further inquiries can be directed to the corresponding author.
